# Neurovascular Unit Dysfunction and Neurodegenerative Disorders

**DOI:** 10.3389/fnins.2020.00334

**Published:** 2020-04-29

**Authors:** Xing Yu, Caihong Ji, Anwen Shao

**Affiliations:** ^1^Department of Surgery, The Second Affiliated Hospital, Zhejiang University School of Medicine, Hangzhou, China; ^2^Department of Neurology, The First Affiliated Hospital, Zhejiang University School of Medicine, Hangzhou, China; ^3^Department of Neurosurgery, The Second Affiliated Hospital, Zhejiang University School of Medicine, Hangzhou, China

**Keywords:** Alzheimer’s disease, blood–brain barrier, neurovascular unit, neurodegenerative disease, target

## Abstract

The neurovascular unit (NVU), composed of vascular cells, glial cells, and neurons, is the minimal functional unit of the brain. The NVU maintains integrity of the blood–brain barrier (BBB) and regulates supply of the cerebral blood flow (CBF), both of which are keys to maintaining normal brain function. BBB dysfunction and a decreased CBF are early pathophysiological changes in neurodegenerative disorders, such as Alzheimer’s disease (AD), Parkinson’s disease (PD), and amyotrophic lateral sclerosis (ALS). In this review, we primarily focus on the NVU in AD as much research has been performed on the connection between NVU dysfunction and AD. We also discuss the role of NVU dysfunction in the pathophysiological mechanisms of PD and ALS. As most neurodegenerative diseases are difficult to treat, we discuss several potential drug targets that focus on the NVU that may inform novel vascular-targeted therapies for AD, PD, and ALS.

## Introduction

Neurodegenerative disorders, like Alzheimer’s disease (AD), Parkinson’s disease (PD), and amyotrophic lateral sclerosis (ALS), are severe neurological disorders that severely affect the quality of life of patients and result in a heavy burden on the economy and society. AD symptoms include memory loss and cognitive impairment (Govindpani et al., 2019). The prevalence of AD patients is high. In 2020, there were approximately 4.7 million people with AD in the United States, and by 2050, the number is expected to triple (Hebert et al., 2013). PD is the second most common neurodegenerative disorder. Common clinical manifestations include tremor, rigidity, and bradykinesia, which result in a heavy burden on patients and society (Lee and Pienaar, 2014). ALS is a common degenerative disease, which affects motor neurons and causes progressive atrophy of skeletal muscles, paralysis, and death (Petrov et al., 2017). However, our understanding of the pathogenesis of these disorders is still limited.

Numerous research studies have demonstrated that the diseases mentioned above are related to disruption of the neurovascular unit (NVU) (Sweeney et al., 2018; Liu et al., 2019). The NVU, which is composed of vascular cells, glial cells, and neurons, plays an important role in maintaining the functional integrity of the blood–brain barrier (BBB) and regulating the volume of the cerebral blood flow (CBF) (Sweeney et al., 2018). Disruption of the NVU may induce dysfunction of the BBB and decrease the CBF, which may contribute to the pathogenesis of neurodegenerative disorders. When the NVU is disrupted and CBF decreases, not only is the supply of oxygen and nutrients to the brain reduced but also the clearance of neurotoxic substance, such as β-amyloid (Aβ) and α-synuclein from brain parenchyma is diminished.

In this review, we will discuss the roles of the NVU in the pathogenesis of neurodegenerative diseases, AD, PD, and ALS. Because the prevalence of Huntington’s disease, multiple sclerosis, and other neurodegenerative diseases is relatively low (McColgan and Tabrizi, 2018), we do not discuss research related to those diseases in this review. We will especially focus on AD because of significant research related to the close interaction between NVU dysfunction and AD. In this article, we describe the composition of the NVU as well as its function in molecular transport and CBF regulation. Moreover, in this study, we review changes in the NVU with respect to the pathogenesis of neurodegenerative disorders, including specific mechanisms in different neurodegenerative diseases. Finally, we review potential therapeutic targets associated with these neurovascular deficits.

## Composition of the Neurovascular Unit

**FIGURE 1:**
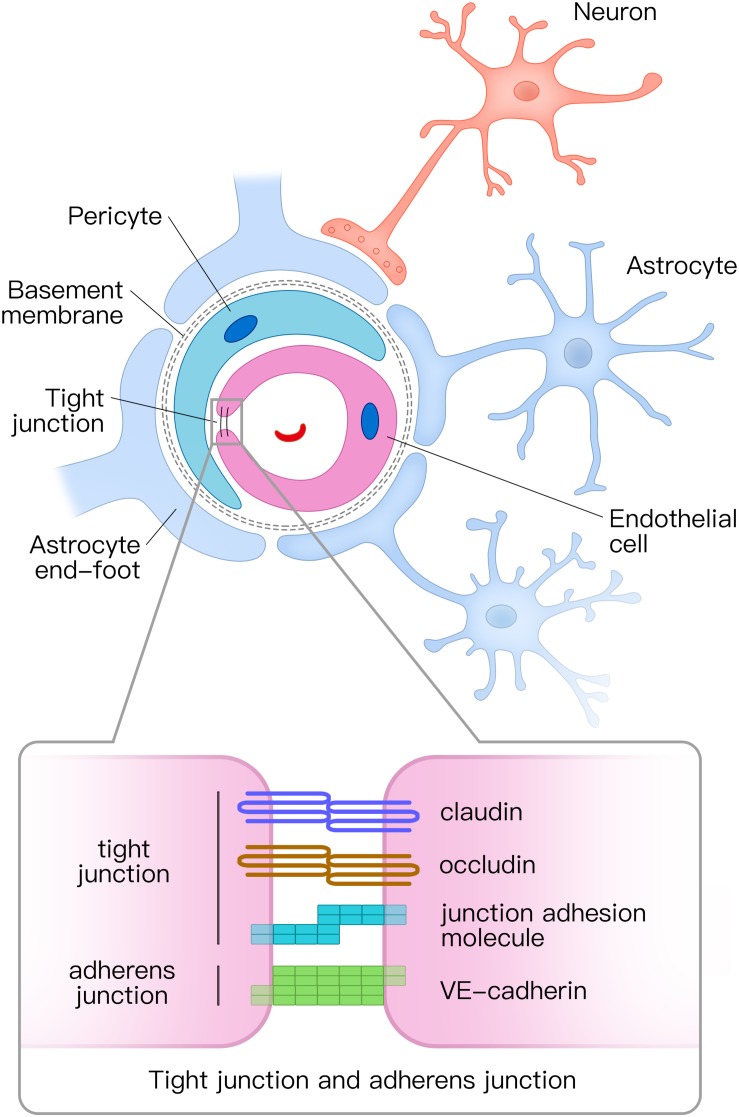
Structural diagram of the neurovascular unit (NVU) and the composition of tight junctions and adherens junctions. The NVU is composed of vascular cells (including endothelial cells, pericytes, and vascular smooth muscle cells), glial cells (astrocytes, microglia, and oligodendroglia), and neurons. Pericytes and astrocyte end-feet surround endothelial tubes. Adjacent endothelial cells are connected by tight junctions and adherens junctions. The tight junction is mainly composed of claudin, occludin, and junctional adhesion molecules, whereas the adherens junction is composed of vascular endothelial (VE) cadherin. NVU, neurovascular unit.

The NVU is composed of vascular cells (including endothelial cells, pericytes, and vascular smooth muscle cells), glial cells (astrocytes, microglia, and oligodendroglia), and neurons (Figure 1; Zlokovic, 2011). The tube structure of the capillaries in the brain is formed by endothelial cells. The outside of the endothelial tubes is surrounded by pericytes and astrocyte end-feet. Moreover, endothelial tubes are surrounded by extracellular matrix that forms the basement membrane. Combined with neurons, all those mentioned above comprise the NVU. Tight junctions and adherens junctions connect endothelial cells and tight junctions limit the paracellular permeability of the BBB (Zlokovic, 2008). There are several transmembrane proteins involved in constructing tight junctions, including claudin, occludin, junctional adhesion molecule, and zonula occludens-1 (ZO-1) (Zlokovic, 2011; Yamazaki and Kanekiyo, 2017). Vascular endothelial (VE) cadherin is the principal cadherin that forms the adherens junction and mediates intercellular adhesion (Zenaro et al., 2017). Both tight and adherens junctions play key roles in the control of endothelial permeability. Tight junctions prevent free diffusion of proteins and seal the paracellular cleft between endothelial cells (Tietz and Engelhardt, 2015), whereas adherens junctions play a key role in cell-to-cell contacts and promote cell maturation (Tietz and Engelhardt, 2015).

Pericytes cover the abluminal surface of capillaries and regulate blood flow by controlling the capillary diameter (Hamilton et al., 2010; Winkler et al., 2011). Pericytes also clear toxic proteins to maintain the stability of the central nervous system (CNS) and play a key role in the formation of tight junctions (Daneman et al., 2010; Sagare et al., 2013).

Astrocytes are the most abundant glial cells in CNS. In the NVU, astrocytes communicate with endothelial cells through their end-feet (Abbott et al., 2006). Astrocytes combine neuronal activity with blood vessels in a process, termed neurovascular coupling. They respond to neuronal activity and deliver signals to regulate the CBF (Attwell et al., 2010; Gordon et al., 2011). Furthermore, astrocytes play a vital role in molecular transport and BBB integrity (Yamazaki and Kanekiyo, 2017).

The NVU is crucial for stabilizing the environment of the brain. Firstly, the continuous endothelial cells with tight junction, basement membrane, and end-feet of astrocytes in the NVU form the BBB. The BBB regulates which molecules or cells enter the brain and clear detrimental proteins from brain parenchyma to the peripheral circulatory system (Yamazaki and Kanekiyo, 2017). Secondly, the NVU regulates the CBF in response to neuronal activity through neurovascular coupling, which ensures sufficient oxygen and nutrient delivery to brain tissue where they are needed (Sweeney et al., 2018).

## Neurovascular Unit Dysregulation in Alzheimer’s Disease

The most widely accepted pathophysiologic mechanism of AD is insoluble Aβ deposition in senile plaques (Vinters, 2015). Aβ accumulation in the brain is highly related to the dysfunction of the NVU (Sweeney et al., 2018) and is thought to be caused by the following two mechanisms, increased permeability of the BBB and reduced CBF.

### Increased Permeability of the Blood–Brain Barrier

As mentioned above, adjacent endothelial cells are connected by tight and adherens junctions, which form the BBB to maintain homeostasis of the brain. Various studies using different experimental methods have shown that the BBB integrity is impaired in AD (Yamazaki and Kanekiyo, 2017).

**FIGURE 2:**
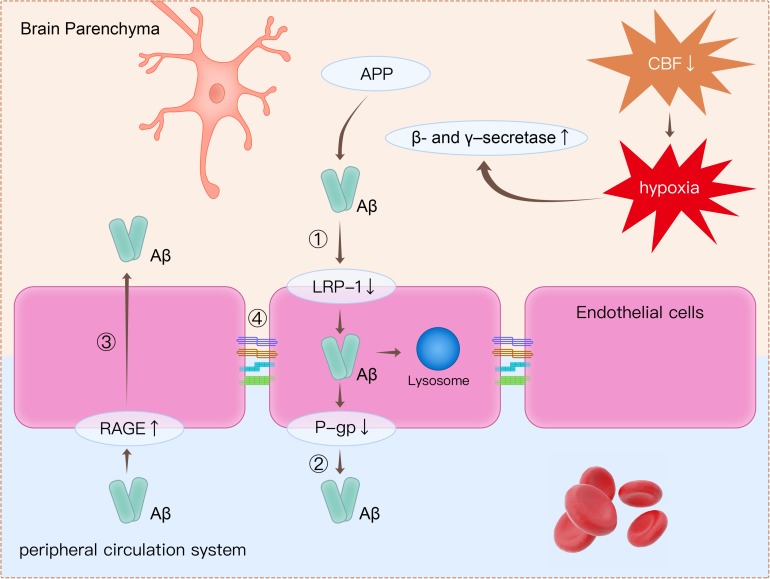
Clearance of β-amyloid (Aβ) from the brain is impaired through several mechanisms. (1) Decreased expression of LRP1 on endothelial cells causes decreased transport of Aβ from the brain to the peripheral circulatory system. (2) P-gp is an ATP-dependent efflux transporter that is expressed in the luminal surface of endothelial cells. Deficient expression of P-gp decreases Aβ clearance. (3) RAGE is an immunoglobulin superfamily member and a receptor for Aβ. Increased expression of RAGE in endothelial cells leads to more influx of Aβ from the peripheral circulatory system to brain parenchyma. (4) Tight junction proteins such as occludin, claudins, and ZO-1 are reduced in endothelial cells, thereby leading to impairment of BBB integrity. Apart from disruption of the BBB, decreased CBF leads to hypoxia, which upregulates the production of β- and γ-secretase. Increased β- and γ-secretase increases the cleavage of Aβ from APP. LRP1, low-density lipoprotein receptor-related protein 1; P-gp, P-glycoprotein; RAGE, receptor for advanced glycation end products; ZO-1, zonula occludens-1; BBB, blood–brain barrier; CBF, cerebral blood flow; APP, amyloid precursor protein.

The detection of plasma-derived proteins in brain parenchyma is an extensively used approach for detecting BBB breakdown. Plasma proteins, including prothrombin, are found in postmortem cortical tissues of AD patients, and leakage of proteins is more common in patients with at least one *APOE4* allele (Zipser et al., 2007). Using a novel dynamic contrast-enhanced MRI protocol to quantify BBB permeability, Montagne et al. (2015) showed that BBB permeability was increased in patients with mild impaired cognitive function than in healthy controls. Furthermore, BBB dysfunction leads to decreased Aβ clearance in AD (Govindpani et al., 2019). There are several mechanisms related to BBB dysfunction, which may lead to amyloid burden in the brain (Figure 2).

Firstly, decreased expression of low-density lipoprotein receptor-related protein 1 (LRP1) and P-glycoprotein (P-gp), together with increased expression of the receptor for advanced glycation end products (RAGE), is are observed in endothelial cells in AD patients (Yamazaki and Kanekiyo, 2017; Zenaro et al., 2017). All these proteins are crucial in Aβ transport across the BBB. LRP1 is expressed on endothelial cells and can internalize Aβ on the abluminal side (Cupino and Zabel, 2014; Yamazaki and Kanekiyo, 2017; Goulay et al., 2019). The internalized Aβ is then transported into lysosome in endothelial cells for further degradation, and some internalized Aβ would be transferred to the luminal side by receptor-mediated transcytosis (Pflanzner et al., 2011; Candela et al., 2015). P-gp is an ATP-dependent efflux transporter that is located on the luminal surface of endothelial cells (Schinkel, 1999). In a previous animal study, it was concluded that deficient expression of P-gp decreased Aβ clearance and increased Aβ deposition in the brain (Cirrito et al., 2005). RAGE is a member of immunoglobulin superfamily and can bind Aβ (Yan et al., 2010). RAGE mediates the entry of Aβ from peripheral vessels to the brain through the BBB. RAGE immunoreactivity in endothelial cells was significantly increased in postmortem AD brains compared with healthy controls (Miller et al., 2008). Increased expression of RAGE in endothelial cells leads to more influx of Aβ from the peripheral circulatory system to brain parenchyma.

Secondly, tight junction proteins such as occludins, claudins, and ZO-1 are reduced in endothelial cells (Marco and Skaper, 2006; Kook et al., 2012; Wan et al., 2015). As reported in previous studies, Aβ was responsible for changes in tight junction protein expression (Marco and Skaper, 2006; Kook et al., 2012; Wan et al., 2015). It has been revealed that Aβ1-42 oligomers disrupt tight junctions and increase permeability of the BBB through reduction in the expression of occludin, claudin-5, and ZO-1 in endothelial cells (Kook et al., 2012; Wan et al., 2015).

### Cerebral Blood Flow Reduction

Decades before the onset of clinical symptoms, CBF in the cortex changed in AD patients (Binnewijzend et al., 2016; Hays et al., 2016; Dong et al., 2018). In AD and mild cognitive impairment patients, arterial spin-labeling MRI demonstrated reduced CBF in temporal and parietal cortices (Schuff et al., 2009; Alexopoulos et al., 2012).

The most widely accepted cause of CBF reduction in AD is the cholinergic-vascular hypothesis (Govindpani et al., 2019). This hypothesis postulates that CBF changes are due to changes in vascular innervation caused by neuronal loss, especially the loss of cholinergic innervation. In a previous study, an extensive reduction in cholinergic neurons in the temporal lobe cortex and hippocampus of postmortem AD brains was shown (Babic, 1999). Cholinergic neurons have a critical role in controlling the vascular tone in the brain (Van Beek and Claassen, 2011). Acetylcholine binding to muscarinic receptors in vascular smooth muscle cells dilates arterioles (Hamel, 2004). The deficit in neurovascular coupling leads to decreased CBF in the brain.

In addition to disruption of neurovascular coupling, vascular abnormalities may lead to decreased CBF in AD (De La Torre, 1997). Vascular abnormalities, such as tortuous, kinking, looping, or twisting arterioles, are common in AD (Baloyannis and Baloyannis, 2012). Such morphological changes in arterioles are due to vessel wall thinning and vascular smooth muscle cells loss. String vessels, which are composed of connective tissue and lack endothelial cells, are not functional in maintaining BBB integrity. The density of string vessels is remarkably increased in the brain gray matter of AD patients (Hunter et al., 2012), and this is related to decreased CBF in the brain.

A decreased CBF also decreases clearance and increases the deposition of Aβ (Mosconi, 2005; Mawuenyega et al., 2010). It has been reported that parenchymal Aβ deposition and cerebral amyloid angiopathy burden are increased in animal models of cerebral hypoperfusion (Garcia-Alloza et al., 2011; Okamoto et al., 2012; Li et al., 2014; Gupta and Iadecola, 2015). Consistent with the data from animal experiments, postmortem human brains showed that the severity of cerebral amyloid angiopathy significantly correlated to cortical microinfarcts (Okamoto et al., 2012). Similar to the role of hypoperfusion in promoting Aβ accumulation, it has been reported that plasma Aβ increased after cardiac arrest in humans (Zetterberg et al., 2011). Hypoperfusion can trigger accelerated deposition of Aβ (Garcia-Alloza et al., 2011). Hypoperfusion in the brain has been shown to dramatically increase the cleavage of Aβ from the amyloid precursor protein (APP), through upregulation of β- and γ-secretase, two enzymes that are required for the production of Aβ (Zhang et al., 2007; Li et al., 2009). Moreover, in addition to increased production of Aβ, decreased CBF leads to insufficient clearance of Aβ. It was found that about half of the Aβ clearance could be attributed to CBF and vascular-perivascular pathways (Roberts et al., 2014).

The effects of brain hypoxia and Aβ deposition are mutual (Gupta and Iadecola, 2015). The accumulation of Aβ deteriorates cerebrovascular function, increases arterial vasoconstriction, and reduces CBF (Thomas et al., 1996; Niwa et al., 2000).

## Neurovascular Unit Dysregulation in Parkinson’s Disease

The widely accepted pathological mechanism of PD involves the loss of dopaminergic neurons in the ventral tier mesencephalon (Lee and Pienaar, 2014). However, it is demonstrated that PD pathology not only is restricted to the dopaminergic system but also influences the noradrenergic, serotonergic, and cholinergic systems (Loane et al., 2013; Pienaar and Van De Berg, 2013). Increasing evidence has shown that disruption of the BBB may play a critical role in the pathological mechanism of PD.

Previous research has revealed that the BBB is disrupted in various toxin-induced PD models, including 6-OHDA and MPTP-treated mice (Carvey et al., 2005; Chen et al., 2008). Other studies have demonstrated that the expression of α-synuclein is associated with increased permeability of the BBB (Jangula and Murphy, 2013). Several mechanisms can explain the BBB disruption in PD patients. Firstly, with increasing age, senile astrocytes and microglia produce various cytokines, chemokines (e.g., IL-6, IL-1β, and TNF-α), and reactive oxygen species (ROS), which disrupt the integrity of the BBB and lead to the rearrangement of tight junctions (Collins et al., 2012). Secondly, PD patients show reduced expression of P-gp in the midbrain, which is related to BBB dysfunction (Kortekaas et al., 2005). It is thought that the decreased P-gp maybe related to an accumulation of α-synuclein and other neurotoxic substances in the brain (Bartels, 2011). Decreased efflux membrane transport through P-gp leads to brain damage due to the accumulation of harmful substances. Furthermore, in PD patients, angiogenesis occurs; however, the newly created vessels are less likely to display the restrictive properties of the BBB. Hence, the poorly developed BBB cannot protect the parenchyma from toxic factors in the peripheral circulation (Desai Bradaric et al., 2012).

## Neurovascular Unit Dysregulation in Amyotrophic Lateral Sclerosis

Amyotrophic lateral sclerosis is a lethal neurological disease involving rapid and progressive degeneration of motor neurons in the brain and spinal cord. Most patients die within 24–48 months after symptom onset (Petrov et al., 2017). A small number of ALS patients have familial ALS, and of those, with a familial etiology, 20% have inherited superoxide dismutase-1 (*SOD1*) mutations, which induce the disease (Garbuzova-Davis et al., 2011). For sporadic ALS, various pathological mechanisms have been proposed; however, the precise pathogenesis is still unclear. One widely accepted pathogenic mechanism of ALS is related to BBB and blood–spinal cord barrier (BSCB) impairment, which leads to motor neuron damage.

Mice with ALS-linked *SOD1* mutations have reduced levels of tight junction proteins, including ZO-1, occludin, and claudin-5, which disrupt the BBB and BSCB functions (Zhong et al., 2008). Human lumbar spinal cords from ALS patients also demonstrate diminished expression of ZO-1 and occludin, which corresponds with the finding in animals (Henkel et al., 2009). Another study showed dramatically reduced perivascular occludin, collagen IV, and astrocyte end-feet surrounded with endothelial cells in the postmortem spinal cord of ALS patients (Miyazaki et al., 2011). Furthermore, the disruption of the BBB and BSCB occurs prior to motor neuron degeneration (Zhong et al., 2008). In the NVU, the degeneration of tight junctions, impairment of endothelial cells, and reduction in astrocytic end-feet contribute to dysfunction of the BBB and BSCB. This leads to vascular leakage and the entry of harmful substances from the peripheral blood into the CNS parenchyma (Garbuzova-Davis et al., 2008). The expression of two proteins important for endothelial cell function, GLUT-1 and CD146, is decreased in ALS (Garbuzova-Davis et al., 2007). Additionally, in *SOD1* mutated mice, blood flow in the cervical and lumbar spinal cord is decreased by about 30–45% before the onset of symptoms (Zhong et al., 2008).

In short, the NVU is impaired in ALS before the onset of clinical symptoms, and damage to the NVU plays a key role in the pathogenesis of ALS (Garbuzova-Davis et al., 2011).

## Implications for Drug Targets

Increasing evidence has shown the important role of the NVU in the pathogenesis of neurodegenerative diseases, including AD, PD, and ALS (Sweeney et al., 2018). Most of these neurodegenerative disorders are intractable, especially AD and ALS. Therefore, looking to vascular cells as potential drug targets for neurodegenerative disorders is a promising avenue of research.

For instance, BBB breakdown and CBF reductions are critical in the pathogenesis of AD. As discussed before, downregulation of LRP1 expression is found in endothelial cells and pericytes of AD patients, and this significantly affects the clearance of Aβ across the BBB. Thus, increasing expression of LRP1 may be a promising therapeutic target for AD patients. Statins can reduce the risk of AD by decreasing Aβ levels. In addition, the molecular mechanism of how statins influence Aβ metabolism involves increasing the expression of LRP1 and accelerating Aβ clearance (Shinohara et al., 2010). Therefore, statins may be recommended for upregulation of LRP1 (Whitfield, 2007). Recent research has found that treatment with 1,25-(OH)2-vitamin D3 increased the expression of LRP1 significantly both *in vivo* and *in vitro* (Patel and Shah, 2017; Cai et al., 2018). Therefore, sufficient vitamin D supplementation may be beneficial for Aβ clearance from the brain parenchyma to blood. Furthermore, loss of cholinergic innervation leads to decreased CBF in AD patients. Acetylcholinesterase inhibitors (AChEIs), like tacrine, galantamine, and donepezil, increase the acetylcholine concentrations and have been widely used for decades (Govindpani et al., 2019). AChEIs have been shown to increase blood perfusion in the frontal lobe and prevent the progression of cognitive impairment after 1 year of treatment (Shimizu et al., 2015).

For abnormal angiogenesis, Aβ immunization therapy in Tg2576 mice has been shown to dramatically reduce the formation of non-functional vessels, which increase vascular permeability and lead to brain damage (Biron et al., 2013). In Aβ-immunized mice, amyloidogenesis-triggered angiogenesis was decreased, and the vascular density reverted to normal levels. However, early clinical trials using the Aβ vaccine in human AD showed unexpected negative side effects. Therefore, additional studies are required prior to the application of Aβ immunization therapy in AD patients (Masliah et al., 2005).

In a mouse model of AD, implantation of encapsulated VE growth factor (VEGF)-secreting cells resulted in increased vascularization and reduced Aβ deposition in the cerebral cortex (Spuch et al., 2010). Furthermore, cognitive behavior improved after implantation of VEGF microcapsules (Spuch et al., 2010). A recent study in mice showed that the transplantation of endothelial progenitor cells into the hippocampus increased microvessel density, whereas the deposition of Aβ senile plaque and hippocampal cell apoptosis was decreased (Zhang et al., 2018). Transplantation of endothelial progenitor cells could also upregulate the expression of the tight junction proteins ZO-1, occludin, and claudin-5 (Zhang et al., 2018).

Given that oxidative stress plays an important role in the underlying mechanism of PD, astrocytes as a part of NVU secrete several beneficial antioxidant compounds, including glutathione (GSH), superoxide dismutases (SODs), and ascorbate (Cabezas et al., 2014). These molecules are important for neuron survival during the neurodegenerative processes (Cabezas et al., 2014). Several studies have showed that GSH was critical for the protection of BBB integrity (Agarwal and Shukla, 1999). Thus, supplementary of these molecules may be an effective treatment for PD.

## Conclusion

In this review, we describe the role of NVU dysfunction in the pathogenesis of several neurodegenerative diseases, including AD, PD, and ALS. BBB breakdown and CBF reduction influence the removal of harmful substances from the brain and play an important role in the onset of neurodegenerative disorders. A focus on the NVU for potential drug targets may be helpful to inform novel vascular-targeted therapies. Nevertheless, the mechanisms of BBB breakdown during neurodegenerative diseases need to be further elucidated.

## Author Contributions

XY and CJ drafted the manuscript. AS reviewed and modified the manuscript. All authors agreed on the final version.

## Conflict of Interest

The authors declare that the research was conducted in the absence of any commercial or financial relationships that could be construed as a potential conflict of interest.
